# Co-Treatment with Human Leukocyte Extract and Albendazole Stimulates Drug’s Efficacy and Th1 Biased Immune Response in *Mesocestoides vogae* (Cestoda) Infection via Modulation of Transcription Factors, Macrophage Polarization, and Cytokine Profiles

**DOI:** 10.3390/pharmaceutics15020541

**Published:** 2023-02-06

**Authors:** Gabriela Hrčková, Terézia Mačak Kubašková, Dagmar Mudroňová, Zuzana Jurčacková, Denisa Ciglanová

**Affiliations:** 1 Institute of Parasitology, The Slovak Academy of Sciences, Hlinkova 3, 040 01 Košice, Slovakia; 2 Department of Microbiology and Immunology, University of Veterinary Medicine and Pharmacy, Komenského 68/73, 041 81 Košice, Slovakia

**Keywords:** albendazole, DLE, treatment, cestode infection, mice, macrophages, monocytes, lymphocytes, peritoneal cavity

## Abstract

The model flatworm *Mesocestoides vogae* proliferating stage of infection elicits immunosuppression in the host. It was used to investigate the effects of human leukocyte extract (DLE) alone and in combination with anthelmintic albendazole (ABZ) on the reduction in peritoneal infection, peritoneal exudate cells (PECs), their adherent counterparts, and peritoneal exudates after the termination of therapy. Balb/c mice were infected with the larvae of *M. vogae*. PECs and adherent macrophages were studied via flow cytometry, mRNA transcript levels, and immunofluorescence. The cytokine levels were measured via ELISA and larvae were counted. ABZ significantly reduced larval counts (581.2 ± 65, *p* < 0.001), but the highest reduction was observed after combined treatment with ABZ and DLE (389.2 ± 119, *p* < 0.001) in comparison with the control. Compared to an infected group, the proportions of CD11b+CD19- myeloid cells with suppressive ability decreased after albendazole (ABZ) in combination with DLE, which was the most effective in the elevation of B cells and CD11b+F4/80^mid/high^MHCII^high^ macrophages/monocytes (22.2 ± 5.4%). Transcripts of the M2 macrophage markers (arginase 1, FIZZ-1, and Ym-1) were downregulated after DLE and combined therapy but not after ABZ, and the opposite trend was seen for iNOS. This contrasts with reduced ex vivo NO production by LPS-stimulated PECs from DLE and ABZ+DLE groups, where adherent macrophages/monocytes had elevated transcripts of the INF-γ receptor and STAT1 and reduced expression of STAT3, STAT6, and IL-10. Each therapy differentially modulated transcription profiles and concentrations of IFN-γ, TNF-α, IL-12p40, IL-6, IL-10, and TGF-β cytokines. DLE strongly ameliorated ABZ-induced suppression of INF-γ and IL-12 and preserved downregulation for IL-4, IL-10, and TGF-β. Epigenetic study on adherent macrophages from infected mice showed that ABZ, ABZ-sulfoxide, and DLE could interact with the mRNA of examined markers in a dose-dependent pattern. Co-administration of DLE with ABZ seemed to augment the drug’s larvicidal effect via modulation of immunity. In comparison with ABZ, combined therapy was the most effective in alleviating parasite-induced Th2/Treg/STAT3/STA6 directed immunosuppression by stimulating the Th1 cytokines, M1 macrophage polarization, and activation of the IFNγ/STAT1 signaling pathway.

## 1. Introduction

The metacestode (larval) stages of zoonotic cestode species such as *Echinococcus multilocularis*, *Echinococcus granulosus*, and *Taenia solium* are causative agents of life-threatening chronic diseases in humans, who represent accidental hosts. The larval stages can grow asexually in different anatomical locations, and behave like benign or malignant tumors with possible metastatic dissemination. It is estimated that several million people have echinococcosis at any time, mostly in endemic areas [1]. The first line of treatment is surgery accompanied by chemotherapy utilizing benzimidazole derivates, such as albendazole (ABZ) and mebendazole [2,3,4,5]. Drugs exhibit relatively good clinical efficacy, but treatment has to last from a few weeks up to life-long. In patients, this aggravates parasite-induced immunosuppression due to the drug´s adverse effects such as bone marrow suppression, reduction in lymphocyte proliferation, and liver toxicity. Moreover, in most cases, drugs can not clear infections caused by tissue-dwelling metacestode parasites with proliferating capacity [2,6], indicating that benzimidazoles are more parasitostatic than parasitocidal [7]. Several novel treatment alternatives are being investigated in various laboratories, mostly focusing on natural products and biologics used as alternatives to the treatment or as adjuvants to the primary therapy [8]. The combination treatment with an immunomodulatory compound, preferably that which is approved for use in human medicine, offers many beneficial effects on the amelioration of immunopathology. One such example is dialyzable leukocytes extracts (DLEs) which are prepared from peripheral blood or tissue leukocytes of healthy human or animal donors. They represent a heterogeneous mixture of low-molecular-weight substances, up to 10–12 kDa of MW. They typically consist of various biologically active components, which include cyclic nucleotides, nicotinamide, purine bases, histamine, ascorbates, prostaglandins, serotonin, amino acids, proteins, etc. [9,10,11,12]. Although the DLEs are non-immunogenic and non-species-specific, they also contain a pool of small peptides called the transfer factor with a molecular weight of 3.5–6.0 kDa to which the oligoribonucleotides are attached [13]. This fraction is responsible for transferring cell-mediated immunity to the particular antigens. The non-immune DLE represents an attractive alternative to complement chemotherapy, which can be used to enhance the immune system impaired by disease and drugs. Dialyzable leukocyte extracts prepared from the blood leukocytes of healthy human donors are available as products under the commercial name IMMODIN^®^ (ImunaPharm, Šarišské Michaľany, Slovakia) and Transferon^®^ (Pharma-FT, ENCB-IPN facilities, Mexico City, Mexico). Recently, the detailed analysis of protein/peptide composition in IMMODIN was performed by Fernando Zuniga-Navarrete et al. [14], who detected forty-eight unique proteins associated with human blood cells or plasma. They were grouped into three classes: molecules associated with the immune response, the inflammatory response, and tissue repair, and a group of proteins involved in regulating cell growth. In the experimental model of 4T1 mouse breast cancer, IMMODIN in combination with paclitaxel [15] or manumycin [16] increased the antitumor effect of drugs, prolonged mice survival, and alleviated the drug´s induced toxicity on immune cells. Other human DLE Transferon^®^ inhibited tumor growth and brain metastasis in a murine model of prostate cancer [17]. In the patients with multidrug-resistant tuberculosis, IMMODIN given as an adjuvant to the chemotherapy contributed to marked improvement of immune parameters in blood, the disappearance of clinical symptoms, and the resorption of infiltrative changes in the lungs [18], and DLEs also showed anti-herpes activity [19]. They positively affected bone marrow hemopoiesis suppressed by ionizing radiation [20].

Helminth parasites, including the larval stages of cestodes, induce an entirely distinct immune response profile from microbial and viral pathogens. In humans and animals, this canonical response is of Th2-type, lately mixed with Treg-type. It predominantly involves IL-4, IL-5, IL-13, and IL-10 TGF-β cytokines as well as the expansion of eosinophil populations, basophils, and mast cells, and alternatively activates the M2-type of macrophages with suppressive functions on different cell types [21,22]. Genes associated with alternatively activated macrophages discretely regulate helminth infection and pathogenesis in mouse models [23,24]. Very little information is available on how albendazole therapy modulates phenotypes and the functions of myeloid-derived cells in patients with metacestode infections.

The metacestode stage of *Mesocestoides (M.) vogae* (syn. *M. corti*) named tetrathyridium is a model cestode parasite that asexually multiplies in rodents and some other vertebrate species. After oral infection, metacestodes migrate to the liver and peritoneal cavity resulting in a chronic infection, while eliciting severe inflammation and liver fibrosis [25,26]. Although rarely infecting humans, *M. vogae* infection shows high biological and immunological similarities with other medically important cestodiases. Mouse infection provides a valuable laboratory model in immunopharmacological studies focusing on the modulation of immunity and pathology by the drugs, natural compounds, and biologicals on the local (peritoneal cavity, liver) and systemic levels.

In the present study, we investigated whether the co-administration of DLE with ABZ can influence the drug’s efficacy against infection via the modulation of myeloid and lymphoid cell compartments. We focused on inflammatory response in the peritoneal cavity of mice infected with tetrathyridia of *M. vogae*, representing a unique biological milieu where proliferating parasites directly interacted with the host effector system and drugs. Here, we showed that combined therapy with DLE and ABZ was the most effective in alleviating parasite-induced Th2/Treg/STAT3/STA6-directed immunosuppression by stimulation of Th1 cytokines, M1 macrophage polarization, and the activation of the IFNγ/STAT1 signaling pathway.

## 2. Materials and Methods

### 2.1. Drugs and Biochemicals

Albendazole (ABZ) was selected as a standard anthelmintic drug (pharmaceutical secondary standard, 100% purity, Sigma-Aldrich Chemical Co., St. Louis, MO, USA). Drug suspension was prepared in 0.1% of cremofore oil in saline for its administration to animals. The DLE used in our study is registered as the immunomodulatory product under the commercial name IMMODIN^®^. This DLE was prepared by pharmaceutical companies SevaPharma, Ltd. (Prague, Czech Republic) and ImunaPharm Ltd. (Šarišské Michaľany, Slovakia) according to the standard protocol described by Cardoso et al. [27] and was provided by the companies for our research purposes. The provided batch was subjected to the tests delineated by European Pharmacopoeia Guidelines and approved by the Slovak and Czech State Institutes for Drug Control. Individual ampoules contain lyophilized extract from 2.0 × 10^8^ leukocytes (defined as one unit) isolated from the peripheral blood of healthy human donors. Each ampoule was dissolved in 4 mL of water for injection before use. RPMI medium (Biochrom, Berlin, Germany) containing 2 mM of stable glutamine was supplemented with 10% heat-inactivated bovine fetal serum (Biochrom, Berlin, Germany), 100 U/mL of penicillin, 100 μg/mL of streptomycin, 10 μg/mL of gentamicin, and 2.5 μg/mL of amphotericin B, and is further termed as complete medium (CM) from here onwards (all from Sigma-Aldrich, St. Louis, MO, USA).

### 2.2. Mice, Infection, and Experiment Design

*Mesocestoides vogae* infection is maintained by a serial passage from infected mice to a naive ICR-strain of mice at the animal facilities of the Institute of Parasitology of the Slovak Academy of Sciences under pathogen-free conditions approved by the State Veterinary and Food Administration of the Slovak Republic, registered under protocol No. 3871/15-221c. Animals were housed in a room with a 12 h light/dark cycle at a temperature of 22 ± 2 °C and were fed with standard chow for laboratory rodents. Feed and water were provided *ad libitum*. In two experiments, male mice (n = 64) were used at the age of 8 weeks. Four healthy mice served as the uninfected control group (Ctrl). The other mice were infected orally with 60–65 tetrathyridia in warm 0.9% NaCl solution obtained from the peritoneal cavity of the infected ICR mouse. Infected mice were divided into four groups, each comprising 7 animals: an untreated control (INF), mice treated with albendazole alone (ABZ), mice treated with DLE alone (DLE), and mice treated with ABZ in combination with DLE (ABZ+DLE). This experiment aimed to study the whole population of peritoneal exudate cells (PECs) and the reduction in larval counts.

The second experiment with the same experimental setting was focused on adherent and non-adherent PEC populations. In this experiment, the reduction in the larval numbers was also investigated. Both compounds were administered daily for 10 consecutive days, from Day 15 to Day 24 post-infection (p.i.). The ABZ was given orally and one dose contained 10 mg of ABZ/kg of body weight, the average daily dose proposed for treating patients with *Echinococcosis* infections [5]. The DLE solution was injected intraperitoneally at a dose of 0.05 U corresponding to a volume of 0.2 mL. This volume’s effects were previously verified in the experiments on mice with two different pathologies [15,20]. The same batch of DLE was used in both experiments. The next day, after the termination of therapy, corresponding to Day 25 p.i., mice were sacrificed and used to collect tetrathyridia, exudates, and peritoneal exudate cells (PECs).

In the pilot pharmacokinetic study (third experiment), infected mice (n = 32) were divided into two groups. The first was treated with ABZ and the second group was treated with ABZ in combination with DLE using the same treatment scheme as in previous experiments. Four healthy mice served as the control. Blood was obtained from mice (4 for each time-point) at the following post-treatment times: 30 min, 2, 4, and 7 h from retro-orbital plexus into heparinized tubes. Plasma was isolated via centrifugation at 408 RCF for 15 min and stored at −80 °C until it was analyzed via LC-MS chromatography to quantify the concentrations of ABZ-SO and ABZ-SO_2_.

### 2.3. Isolation of Exudates and Cell Sample Preparation

Exudates from peritoneal cavities of healthy and infected mice were collected via washing the peritoneal cavity with 1 mL of sterile PBS. After removing the PECs, exudates were stored at −80 °C for cytokine analysis. Cells obtained after the second peritoneal lavage with 5 mL of CM were pooled with cells from the first lavage. The viability and numbers of isolated cells were evaluated via the trypan blue exclusion test (Sigma-Aldrich, St. Louis, MO, USA) and cells were used for further analyses. The aliquots of PECs were immersed in 1 mL of RiboZol reagents (VWR Chemicals, Fountain Parkway, Solon, OH, USA) and frozen at −80 °C until used for the RNA extraction. Then, larvae were obtained after washing peritoneal cavities with saline.

The second experiment aimed to analyze adherent and non-adherent populations of peritoneal cells. As described above, PECs and peritoneal exudates were isolated from each mouse/group. After cell counting, 5 × 10^6^ PECs/mouse/group were re-suspended in CM medium and plated into 6-well Falcon culture plates (Corning Incorporated, OneRiverfront Plaza, NY, USA), and allowed to adhere for 2 h at 37 °C. Separation was carried out utilizing the ability of macrophages/monocytes to adhere to the plastic surfaces. Then, non-adherent cells were removed by gently washing three times with warm DPBS (Sigma-Aldrich, St. Louis, MO, USA) and adherent cells were detached using warm StemProR Accutase cell Dissociation Reagent (Gibco, Life Technologies Corporation, Grand Island, NE, USA) according to the instructions. After washing, viability determination, and counting, cells were used for the phenotypic analysis via flow cytometry and RNA extraction.

### 2.4. In Vitro Experiments on Adherent PECs from Infected Mice

Additionally, for in vitro epigenetic analysis, adherent cells from infected mice were pooled and plated into a 75 cm^2^ tissue culture flask (Greiner bio-one, Frickenhausen, Germany) and adherent cells were obtained as described above. Then, 2 × 10^6^ adherent cells were plated into 24-well plates in triplicates/treatment in 1 mL of CM medium and treated with the following compounds at the following concentrations: ABZ (0.5, 1.0, 2.0 μg/mL), albendazole sulfoxide (ABZ-SO) (0.025, 0.05, 0.1 μg/mL), purchased from Sigma and dissolved in DMSO. The DLE was used at 50, 100, and 200 µL/mL concentrations. Moreover, the effects of the selected combination of drugs were examined (DLE 100 µL/mL + ABZ 2 µg/mL; DLE 100 µL/mL + ABZ-SO 0.1 µg/mL). Cells were incubated at 37 °C, 5% humidity for 72 h, and washed, and RNA was isolated as described in the next paragraph.

### 2.5. RNA Isolation and Real-Time PCR

In the populations of whole peritoneal cells or adherent PECs, real-time PCR (RT-PCR) was applied to determine the relative quantities of mRNA. The expression of genes for cytokines IFN-γ, TNF-α, IL-12p40, IL-6, IL-4, TGF-β, and IL-10, transcription factor NF-κB, and macrophage markers arginase 1, FIZZ-1, Ym-1, and iNOS was evaluated in total PECs. Adherent macrophages/monocytes were analyzed for mRNA abundance of transcription factors STAT1, STAT3, and STAT6, IFN-γ receptor (IFN-γR), IL-12p40, and IL-10. Moreover, mRNAs isolated from adherent PECs from infected mice after cultivation with ABZ, ABZ-SO, and DLE were examined for the expression of IFN-γ, IL-12p40, IL-10, arginase 1, iNOS, NF-κB, and IFN-γ receptor as previously described [28]. Total RNA was extracted from the cell using RiboZol reagent (VWR Life Science, Radnor Corporate Center, Radnor, PA, USA). Quantitative PCR analysis of the relative abundance of mRNA species was determined using the iTaq SYBR green master mix (BioRad, Hercules, CA, USA) on a BioRad CFX thermocycler (BioRad, Hercules, CA, USA). Relative mRNA expression was calculated by comparative quantification cycle (Cq), normalized to housekeeping gene β-actin utilizing the 2^−∆∆Ct^ method. The list of primers and their sequences are shown in Table S1 (Supplementary Materials).

### 2.6. Staining of Adherent PECs

To evaluate morphology and protein expression of intracellular proteins arginase 1, iNOS, and STAT1, the samples of adherent PECs from experimental groups were cultured on Tissue Culture Treated Glass Slides (Falcon, Corning Brand, County Route, NY, USA) for 2 h. Some slides were fixed in 75% methanol in PBS, stained with May–Grünwald/ Giemsa solutions (Sigma-Aldrich, St. Luis, MO, USA), and used for morphological evaluation. Approximately 200 cells were counted on each slide/mouse/group and the proportions of spindle-like and round-like cells were calculated as mean ± SD (in %) from total cells. The stained cells were observed under a light microscope (Olympus BX-51, Tokyo, Japan). Cell samples from the untreated group were fixed in 4% paraformaldehyde and used for immunocytochemical detection of proteins via fluorescent microscopy. After permeabilization with 0.5% Triton X-100 in PBS and blocking with 2% BSA, cells were incubated with primary antibody against arginase 1, iNOS (both rabbit polyclonal IgG) (Abcam, Cambridge, UK), and STAT1 (mouse monoclonal IgG, purified) (rabbit polyclonal IgG) (Santa Cruz Biotechnology, Dallas, TX, USA) overnight at 8 °C. Slides were then incubated with the secondary antibodies (FITC-conjugated goat anti-rabbit IgG, or FITC-conjugated rabbit anti-mouse IgG, 1:500, both from R&D Systems, Minneapolis, MN, USA) in the dark for 1 h at room temperature. Nuclei were stained using Draq5 dye (1:1000, Abcam, Boston, MA, USA). Images were obtained using a Leica TCS SP5X confocal fluorescent microscope and LAS software (Leica Microsystems, Mannheim, Germany).

### 2.7. Flow Cytometric Analysis

Proportions of myeloid cells were analyzed via fluorescence-activated cell sorting (FACS). Aliquots of whole PECs and adherent macrophages (0.5 × 10^6^ cells/100 μL) were first incubated with anti-CD16/anti CD32 antibodies to block non-specific binding followed by incubation with specific fluorochrome-conjugated monoclonal antibodies. Total PECs were labeled with anti-CD11b (FITC; clone M1/70, Biolegend, San Diego, CA, USA) and anti CD19 (PE; clone 1D3, eBioscience, San Diego, CA, USA) to exclude B cells, which also bear the CD11b marker. This gating strategy allowed us to identify the population of mononuclear myeloid-derived cells. In addition, the PECs were stained with F4/80 (APC; clone CI: A3-1, BioRad, Hercules, CA, USA) and MHCII (PE-Cyanine7; clone M5/114.15.2, eBioscience). Cells gated as CD11b+CD19-F4/80+MHCII+ were further gated on the populations CD11b+F4/80^mid^MHCII^low^ and CD11b+F4/80^mid/high^MHCII^high^. Lymphoid populations were detected after staining with antibodies for T cell receptor CD3 (FITC; clone17A2, eBioscience) and transmembrane protein CD19, which is present on B cells, except for plasma cells. In the second experiment, adherent PECs were stained with antibodies to CD11b, F4/80 and CD19 to verify the absence of B cells. Staining of the cells with antibodies for 30 min at room temperature was followed by washing steps prior to the analysis via flow cytometry. Phenotypic analysis was performed using the FACS Canto cell analyzer (Becton Dickinson Biosciences, Franklin Lakes, NJ, USA). The acquired data were analyzed using the FACS Diva software.

### 2.8. Determination of Nitrite Production by Peritoneal Cells Ex Vivo

To measure the production of NO by PECs ex vivo, cell suspensions of peritoneal cells (1 × 10^6^ cells/mL) from each mouse/group were cultured in 24-well plates (Corning Incorporated, OneRiverfront Plaza, NY, USA Corning) in CM in the presence or absence of lipopolysaccharide (LPS) (Sigma-Aldrich, St. Louis, MO, USA) (1 µg/mL). The plates were incubated for 72 h at 37 °C with 5% CO_2_. The NO concentration in the culture supernatants (50 µL) was determined as nitrite (NO_2_^−^) using Griess reagent. Nitrite concentration was determined from the standard curve of 0.1 M NaNO_3_ dilutions.

### 2.9. Cytokine Detection in Mouse Peritoneal Exudates

The concentrations of IFN-γ, TNF-α, IL-12, IL-6, IL-4, TGF-β, and IL-10 in the peritoneal fluid were quantified using the commercial ELISA Kits (Mouse Ready-SET-Go ELISA, all from eBioscience, San Diego, CA, USA) according to the manufacturer’s instructions. Cytokines were determined individually for each animal/group (n = 7) in mice from the first experiment. Concentrations of cytokines were calculated in pg/mL from the standard curves.

### 2.10. Preparation and Analysis of Standard ABZ and Its Metabolites via LC-MS

First, as the internal standard, anthelmintikum mebendazole (MBZ) was selected [29] and 2.5 μM stock solution was prepared in DMSO: methanol (1:1). Plasma samples (50 μL) from each mouse/group (n = 4) were lyophilized and dry powder was dissolved in 50 μL of MBZ solution to avoid the excessive dilution of metabolites. Following centrifugation at 9000 RCF for 3 min, the plasma samples were diluted in 50 μL of DMSO: methanol (1:1) and used for analysis. The percentage of ABZ-SO and ABZ-SO_2_ recovery in plasma from treated mice was verified via the samples from healthy mice (n = 3). Fifty μL of samples was spiked with one of the metabolites before lyophilization to obtain 1.25 μM of the final concentration, and was further processed as described previously. In order to achieve the selectivity, four blank samples from infected untreated mice were analyzed. Spectral interference of heparin was evaluated since it was used as the anticoagulant in blood samples. Solutions of ABZ and its metabolites were also prepared to measure the retention time. Accuracy of this method was assured with the extraction of five spiked samples of 200 ng/mL of ABZ-SO and 20 ng/mL of ABZ-SO_2_.

### 2.11. Chromatographic Conditions

Mass spectra were obtained using the Shimadzu Prominence system, consisting of a DGU-20A_3_ mobile phase degasser, two LC-20AD solvent delivery units, the SIL-20AC cooling auto sampler, a CTO-10AS column oven, an SPD-M20A diode array detector, and an LCMS-2020 mass detector with single quadrupole equipped with an electrospray ion source (Shimadzu, Kyoto, Japan). Binary gradient elution was used: mobile phase A = 5% acetonitrile in water, 0.1% formic acid; mobile phase B = 80% acetonitrile in water, 0.1% formic acid; gradient: 0–3 min 0–40% B, 3–5 min 40–70% B; 5–7 min 70% B, 7–9 min 70–0% B, 9–10 min 0% B. The flow rate was 0.4 mL/min at 25 °C, injection volume was 10 μL, and samples were detected at 285 nm. Retention times (min) were the following: albendazol (6.811), albendazol sulfoxide (5.328), mebendazol (9.945), albendazol sulfoxide (6.206). The MS parameters were as follows: positive mode; ESI interface voltage, 4.5 kV; detector voltage, 1.15 kV; nebulizing gas flow, 1.5 mL.min^−1^; drying gas flow, 15 mL.min^−1^; heat block temperature, 200 °C; DL temperature, 250 °C; SCAN mode 200–400 m/z; software LabSolutions ver. 5.75 SP2.

### 2.12. Metacestode Burden in the Peritoneal Cavity

The larvae collected from the peritoneal cavities of infected and treated mice were re-suspended in 0.1% agar solution to prevent the sedimentation of larvae and counted under a light microscope. The effect of chemotherapy was assessed by comparing the larval counts in treated versus untreated mice from both experiments, and values (n = 14) are expressed as mean ± SD.

### 2.13. Statistical Analysis

Data obtained from individual analyses for the indicated number of samples were finally calculated as means ± SD. Results were analyzed by one-way ANOVA followed by Tukey’s post hoc test. In the grouped analyses, two-way ANOVA was used followed by Bonferroni’s multiple comparisons or the Sidac post hoc test to compare differences between indicated groups. Differences were regarded as significant for *p* < 0.05, *p* < 0.01, and *p* < 0.001. Data were evaluated using GraphPad Prism (version 7) (GraphPad Software, Inc., San Diego, CA, USA).

## 3. Results

### 3.1. Proportions of Myeloid Peritoneal Exudate Cells

**Figure 1:**
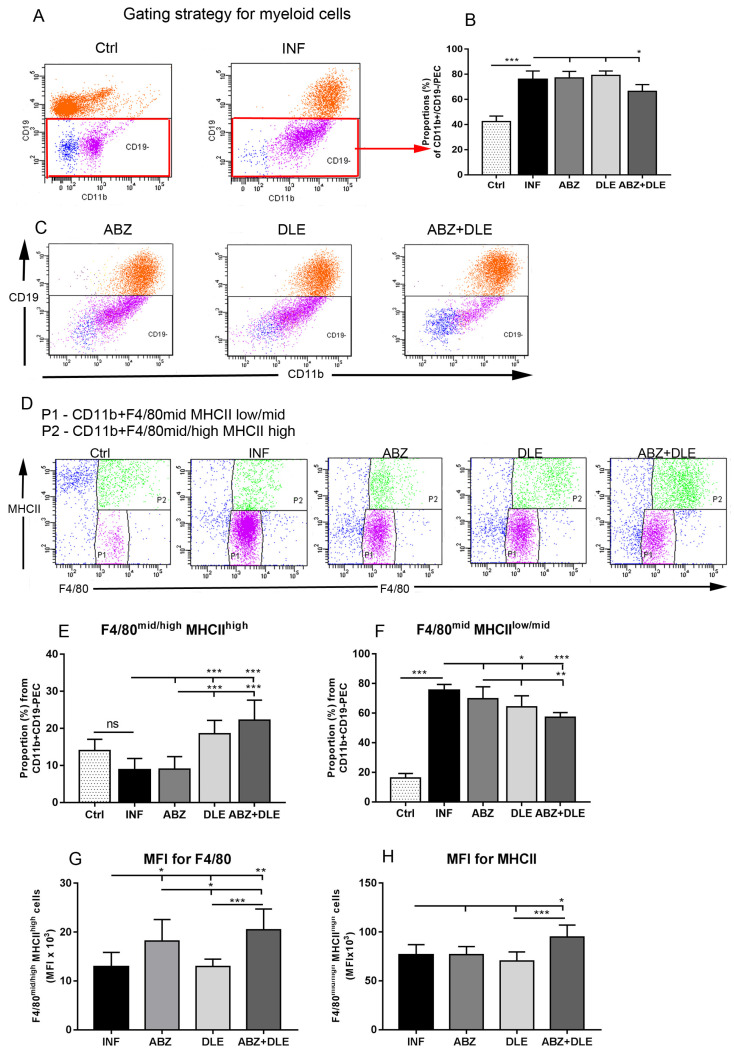
Phenotypic analysis of CD11b+ peritoneal exudate cells (PECs) isolated from control healthy mice (Ctrl), *M. vogae*-infected mice (INF), and infected mice treated with albendazole (ABZ), DLE, and their combination (ABZ+DLE) was performed using flow cytometry; (**A**) the gating strategy on CD11b+CD19- peritoneal myeloid cells in healthy and infected mice; (**B**) the proportions (%) of these cell populations in individual groups of mice (n = 7/group); (**C**) representative dot plots of the gating strategy of CD11b+CD19- myeloid cells in treated groups; (**D**) representative dot plots showing expression of F4/80 and MHCII on CD11b+CD19- myeloid cells; (**E**) the proportions of F4/80^mid/high^MHCII^high^ cells; (**F**) the proportions of F4/80^mid^MHCII^low/mid^ cells within CD11b+CD19- cells in individual groups; (**G**) mean fluorescence intensity (MFI) of F4/80; and (**H**) MHCII expression on CD11b+CD19- cells gated on F4/80^mid/high^MHCII^high^ markers. Significantly different values between individual groups are showed by connecting lines and are marked as * *p* < 0.05, ** *p* < 0.01, and *** *p* < 0.001.

We and other studies showed a rapidly elevating number of inflammatory cells with progressing parasite proliferation in the peritoneal cavities of ICR and C57BL/6 mice strains [26,30]. Peritoneal exudate cells (PECs) were isolated from healthy, infected, and treated mice, and the proportions of myeloid and lymphoid populations were analyzed using flow cytometry. CD11b is the surface marker on myeloid cells (dendritic cells, macrophages, monocytes, neutrophils, eosinophils) and was also detected on B lymphocytes. Peritoneal exudate cells were stained with antibodies to CD11b, CD19, F4/80, and MHCII markers, and the proportions of myeloid CD11b+CD19- cells (Figure 1A—gating strategy for healthy and infected mice) are shown in Figure 1B. They represented 42.3 ± 4.5% in healthy mice and 75.8 ± 6.8% in infected mice, and the significant decline was seen after ABZ+DLE therapy (66.4 ± 5.4%, *p* < 0.05). The representative dot plots are seen in Figure 1C. We further examined CD11b+CD19- macrophage/monocyte populations gated as F4/80^mid^MHCII^low/mid^ and F4/80^mid/high^MHCII^high^ cells. The F4/80 membrane protein is the best-known and most broadly used mouse macrophage marker and the MHCII receptor (major histocompatibility complex) is expressed on most myeloid cells. Figure 1D shows the representative dot plots from all experimental groups. The less abundant was the population of F4/80^mid/high^MHCII^high^ macrophages/monocytes and this significantly increased after DLE and more after ABZ+DLE therapy (22.2 ± 5.4%, *p* < 0.001, Figure 1E) in comparison with infected mice (8.76 ± 3.1%). Populations of F4/80^mid^MHCII^low/mid^ PECs (75.4 ± 39% in infected mice) significantly declined after DLE (64.2 ± 7.4%, *p* < 0.05) and more after combined therapy (57.17 ± 3.2%, *p* < 0.001) (Figure 1F), and for ABZ-treated mice, suggesting that combined therapy preferentially stimulated the accumulation of macrophages/monocytes with high antigen-presenting capacity. The expression of the F4/80 receptor within F4/80^mid/high^MHCII^high^ cells determined as MFI (mean fluorescence intensity) was significantly upregulated in mice treated with ABZ and ABZ + DLE (*p* < 0.01) (Figure 1G), but MHCII expression was elevated after combined therapy only (*p* < 0.01) in comparison with infected mice (Figure 1H). Data indicate the synergistic effect of both drugs on the stimulation of macrophage/monocyte antigen-presenting capacity.

### 3.2. Proportions of Lymphoid Peritoneal Exudate Cells

**Figure 2:**
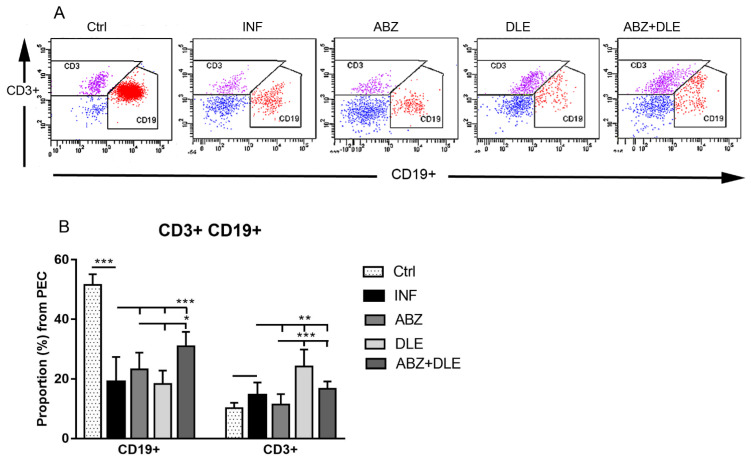
Phenotypic analysis of lymphoid peritoneal exudate cells (PECs) isolated from control healthy mice (Ctrl), *M. vogae*-infected mice (INF), and infected mice treated with albendazole (ABZ), DLE, and their combination (ABZ+DLE) was performed using flow cytometry. (**A**) The representative dot plots gated on CD3+CD19+lymphoid peritoneal cells in individual groups of mice. (**B**) The mean proportions (%) of these cell populations in individual groups of mice (n = 7/group). Significantly different values between individual groups are shown by connecting lines and marked as * *p* < 0.05, ** *p* < 0.01, and *** *p* < 0.001.

The lymphoid population in peritoneal cavities was further analyzed for the presence of B lymphocytes (CD19+CD3-) and T lymphocytes (CD3+CD19-) (Figure 2A, representative dot plots; Figure 2B, proportions of cells). Proportions of B cells significantly declined in infected mice in comparison with healthy mice (19.0 ± 8.3% vs. 51.5 ± 3.6%, *p* < 0.001) and elevation was observed after combined therapy (30.9 ± 4.9%, *p* < 0.001), probably due to stimulated proliferation (not shown). T lymphocytes formed the smaller population in infected mice (14.5 ± 4.3%) and their proportions significantly increased only after DLE administration (24.1 ± 5.7%, *p* < 0.01).

### 3.3. Gene Expression of Myeloid Cell Markers in Peritoneal Exudate Cells

**Figure 3:**
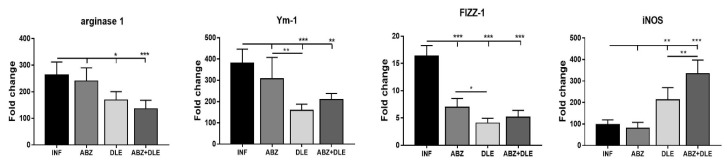
Relative gene expression of mRNA encoding arginase 1, FIZZ-1 (found in inflammatory zone), Ym-1 (chitinase-like protein 3), and iNOS (inducible nitric oxide synthase) markers was analyzed in the peritoneal exudate cell (PEC) samples from each mouse (n = 7/group), and data represent means ± SD. RNA was isolated from PECs after the termination of therapy and relative gene expression was calculated via the 2^−∆∆Ct^ method, using data for control healthy mice as the calibrator. Significantly different values between selected groups are indicated as * *p* < 0.05, ** *p* < 0.01, and *** *p* < 0.001.

Several putative effector molecules have been widely used to distinguish macrophages associated with Th1 immunity characterized as pro-inflammatory M1-type and anti-inflammatory/regulatory M2-type macrophages with suppressive activity. As opposed to M1 macrophages which have high inducible nitric oxide synthase (iNOS) activity, M2 macrophages demonstrate increased arginase 1 activity, and high levels of FIZZ-1 and Ym-1 molecules, which belong to the chitinase family [31,32]. We further characterized the effect of infection and therapy with ABZ, DLE, and their combination on the mRNA levels of these markers (Figure 3). The expression of genes for arginase 1, FIZZ-1, and Ym-1 was highly upregulated in PECs isolated from infected mice (*p* < 0.001) and only moderately upregulated for iNOS in comparison with healthy mice. Albendazole therapy significantly downregulated the expression of genes for FIZZ-1 (*p* < 0.001), but not for arginase 1 or Ym-1. The administration of DLE alone and combination therapy significantly lowered mRNA levels for all three M2 markers, whereas they stimulated gene transcription for iNOS (*p* < 0.001). Data suggest that molecules present in this human non-immune DLE can modulate the activity of genes, regulating the functional properties of macrophages in Th2- biased immunity towards more parasitocidal M1-type, and can abolish the suppressive effect of ABZ on iNOS expression.

### 3.4. m-RNA Transcription Profiles of Cytokines and NFκB in Peritoneal Exudate Cells

**Figure 4:**
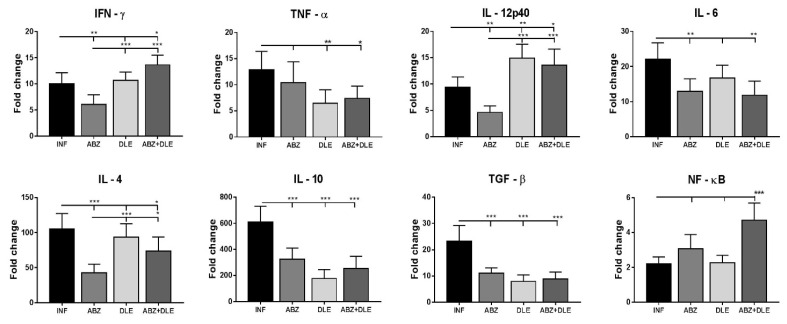
Relative mRNA expression of cytokines IFN-γ (interferon gamma), TNF-α (tumor necrosis factor alpha), interleukins IL-12p40, IL-6, IL-4, and IL-10, TGF-β (transforming growth factor beta), and transcription factor NF-κB (nuclear factor kappa B) was analyzed in the samples of peritoneal exudate cells (PECs) from each mouse (n = 7/group). Data represent means ± SD. RNA was isolated from cells after the termination of therapy corresponding to Day 25 p.i. and relative gene expression was calculated via the 2^−∆∆Ct^ method, using data for control healthy mice as the calibrator. Significantly different values between indicated groups are shown as * *p* < 0.05, ** *p* < 0.01, and *** *p* < 0.001.

Cytokines produced by lymphoid and myeloid cells during helminth infection are key players in orchestrating inflammatory responses from the initial Th1-type to the Th2-type and lately the Treg-type, which are linked to the chronic stage of infection [22]. Interestingly, the administration of ABZ downregulated gene transcription for all of the cytokines in comparison with infected mice (Figure 4). A significant effect of DLE therapy was the stimulation of IL-12p40 (*p* < 0.001) and the suppression of TNF-α (*p* < 0.05), IL-10 (*p* < 0.001), and TGF-β (*p* < 0.001), compared to infected mice. In the group which received combined therapy, both drugs seemed to specifically modulate the final effects at the mRNA levels, and the most profound changes were the significant stimulation of IFN-γ (*p* < 0.001) and IL-12p40 (*p* < 0.01) in parallel with the suppression of IL-10 and TGF-β (*p* < 0.001). Nuclear transcription factor κB (NF-κB) is involved in the innate and adaptive immune response. In our study, mRNA transcripts were significantly elevated only after combination therapy (*p* < 0.001).

### 3.5. Cytokine Concentrations in the Peritoneal Exudates

**Figure 5:**
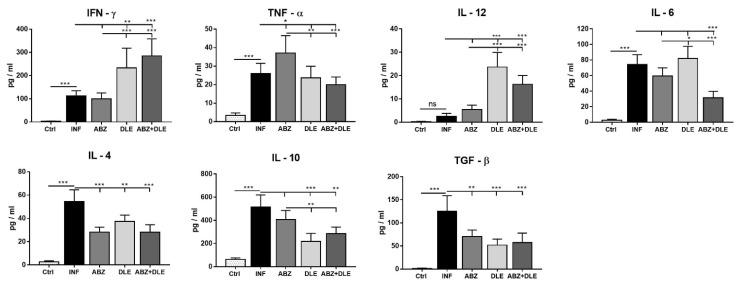
Concentrations of cytokines: IFN-γ, TNF-α, IL-12, IL-6, IL-4, IL-10, and TGF-β, determined via ELISA assays in the peritoneal exudates (1 ml) isolated from control healthy mice (Ctrl), *M. vogae*-infected mice (INF), and infected mice treated with albendazole (ABZ), DLE, and their combination (ABZ+DLE). Data are presented as the mean concentration (n = 7) ± SD (pg/mL). Significantly different values between selected groups of mice are indicated as * *p* < 0.05, ** *p* < 0.01, and *** *p* < 0.001; ns = not significant.

We also determined cytokine concentrations using ELISA Kits in the peritoneal exudates collected by the lavage of cavities with 1 mL of DPBS (Figure 5). In control mice, except for IL-10, levels of pro-inflammatory and anti-inflammatory cytokines were very low. Infection-elicited inflammatory cells produced significantly higher cytokine levels, except for IL-12 (*p* < 0.001). Compared with this group, ABZ administration resulted in moderately increased TNF-α, whereas it suppressed the production of IL-4 and TGF-β (*p* < 0.001) and only slightly decreased IL-10 production. Within this treated group, we noticed a discrepancy between low gene expression and elevated cytokine levels of TNF-α. The possible explanation for this is that the additional source of this cytokine in the peritoneal cavity could be the epithelial cells. In infected mice, DLE administration strongly stimulated IFN-γ and IL-12 production by peritoneal cells (*p* < 0.001) and reduced the secretion of IL-4, IL-10, and TGF-β.

### 3.6. Production of NO by Peritoneal Cells Ex Vivo

**Figure 6:**
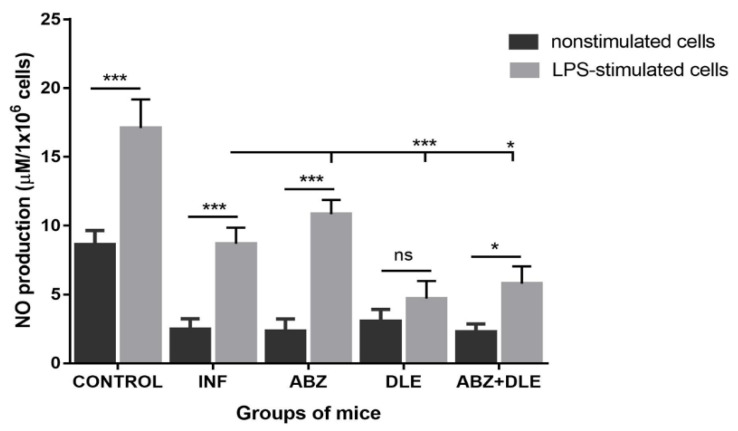
The concentration of NO determined ex vivo in the culture supernatants of unstimulated and LPS-stimulated adherent PECs (1 × 10^6^) isolated from control healthy mice, *M. vogae*-infected mice (INF), and infected mice treated with albendazole (ABZ), DLE, and their combination (ABZ+DLE). NO was determined as nitrite (NO_2_) using a Griess reagent. Significantly different values between indicated groups are indicated as * *p* < 0.05 and *** *p* < 0.001; ns—non significant.

Next, we examined NO production by enzyme iNOS, which utilizes L-arginine as a substrate and competes for the same substrate with arginase. Adherent PECs were incubated for 72 h ex vivo and NO was determined in non-stimulated and LPS-stimulated cells (Figure 6). Resident peritoneal macrophages from healthy control mice produced higher levels of NO in unstimulated and LPS-stimulated cultures when compared with populations of cells from infected mice, indicating that macrophages/monocytes possess the reduced ability to respond to LPS stimulation. Significantly higher NO levels were detected in supernatants of LPS-stimulated cells in all groups, except for DLE-treated mice, where the most significant decline in NO (4.71 ± 1.26 µM) was found in comparison with the infected group (*p* < 0.001). Unstimulated cells from all experimental groups produced very low levels of NO ex vivo and moderate elevation was found in the DLE-treated group.

### 3.7. Morphological and Phenotypic Analysis of Adherent Peritoneal Cells

**Figure 7:**
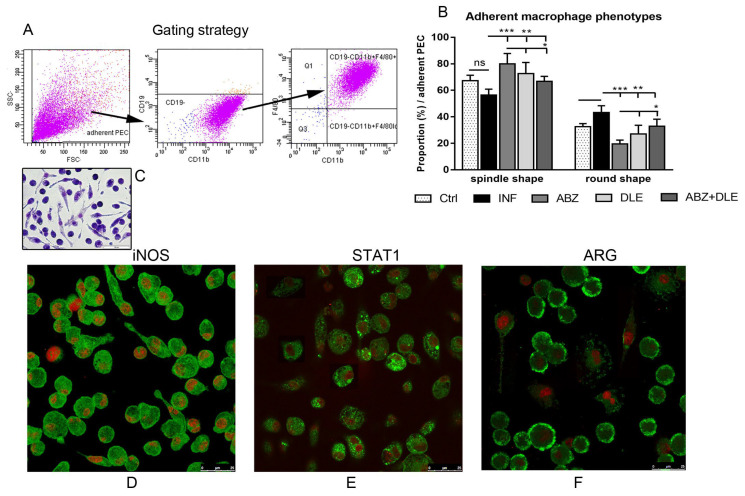
The phenotypic analysis of peritoneal adherent macrophages obtained from control healthy mice (Ctrl), *M. vogae*-infected mice (INF), and infected mice treated with albendazole (ABZ), DLE, and their combination (ABZ+DLE). (**A**) The gating strategy for analysis of CD11b+CD19-F4/80+ monocytes/macrophages determined via flow cytometry. Arrows indicate the steps how gating was performed; (**B**) the proportions of morphologically distinct populations of adherent cells, the spindle-like type and round type; (**C**) the representative image of adherent peritoneal cells after May–Grünwald staining (scale bar = 50 μm); the representative images of adherent PECs from infected untreated mice after immunofluorescent staining for iNOS (**D**), STAT1 (**E**), and arginase 1 (**F**), showing different patterns of marker localization in the cells (red—nuclei, green—cytoplasmic proteins). Significantly different values between individual groups are indicated as * *p* < 0.05, ** *p* < 0.01, and *** *p* < 0.001; ns—not significant.

The effect of therapy with ABZ and other anthelmintic drugs on the modulation of phenotypes and functions of macrophages and recruited monocytes in cestode infections is poorly understood. Therefore, in the next experiment we focused on populations of adherent peritoneal macrophages/monocytes. Flow cytometric analysis of isolated adherent cells from experimental groups confirmed that CD11b+CD19-F4/80+ macrophages’/monocytes’ purity was more than 95% (not shown) (Figure 7A, gating strategy). Adherent cells formed one discrete population via flow cytometry; however, following adherence on glass slides, we observed two morphologically distinct populations, the spindle-like type and round-like type (Figure 7C). In our study, microscopic observations revealed the highest differences in the ABZ-treated group (*p* < 0.001) (Figure 7B) in comparison with the untreated group. The significant decline in spindle-like cells and the elevation of round cells was found following ABZ+DLE administration (*p* < 0.05). We further characterized both phenotypes for the immunoreactivity to iNOS, STAT1, and arginase 1, and representative images from the untreated group are shown in Figure 7D–F. The presence of examined proteins was detected in all adherent cells with very similar distribution in spindle and round cells, and differences were seen in markers’ localization. However, iNOS was homogeneously localized through cytoplasm, arginase was localized mostly below the membrane, and STAT1 formed discrete clusters of proteins.

### 3.8. Gene Expression of Selected Markers in Adherent Macrophages

The activation status of adherent macrophages was further characterized by the transcriptional level of the IFN-γ receptor (IFN-γR), Th1 cytokine IL-12p40, regulatory cytokine IL-10, and transcription factors “signal transducer and activator of transcription” STAT1, STAT3, and STAT6. The activation of STATs is a hallmark of innate as well as adaptive immune response. M2 macrophages are important producers of IL-10 cytokine.

**Figure 8:**
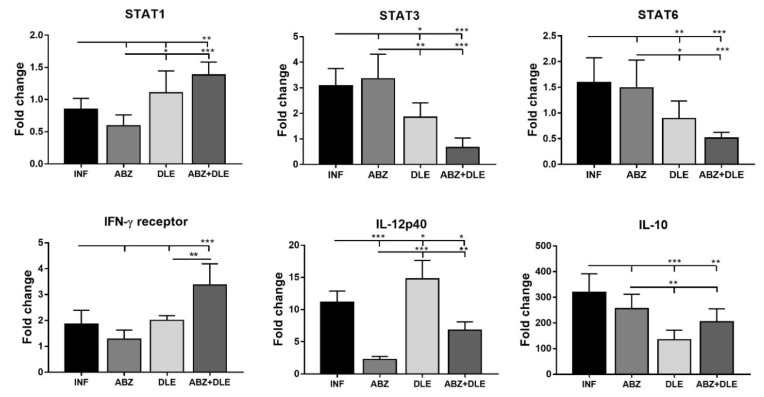
Relative mRNA expression of transcription factors “signal transducer and activator of transcription” STAT1, STAT3, and STAT6, IFN-γ receptor, and cytokines IL-12p40 and IL-10 was determined in the samples of peritoneal adherent macrophages from each mouse /group. Data represent means ± SD (n = 7). RNA was isolated from the cells after the termination of therapy corresponding to Day 25 p.i. and relative gene expression was calculated via the 2^−∆∆Ct^ method, using data for control healthy mice as the calibrator. Significantly different values between selected groups are indicated as * *p* < 0.05, ** *p* < 0.01, and *** *p* < 0.001.

As shown in Figure 8, mRNA transcripts for all examined genes, except for IL-12p40, were not significantly modulated following ABZ therapy in comparison with infected mice. A significant stimulation of gene activity for IFN-γR and STAT1 in parallel with the suppression of STAT3, STAT6, and IL-10 was seen in the groups treated with DLE and ABZ+DLE. IL-12p40 is produced primarily by activated inflammatory cells including macrophages, neutrophils, dendritic cells, and others, when stimulated by pathogens and inflammatory stimuli [33]. Here, in contrast with the INF-γ pathway, the infection activated gene transcription for IL-12p40 compared to the healthy mice. The administration of ABZ alone and combined with DLE downregulated gene transcription for IL-12p40, while the administration of DLE therapy moderately elevated mRNA copies (*p* < 0.05) compared to infected mice. Significantly downregulated IL-10, STAT3, and STAT6 gene transcription after DLE and combination therapy correlated with the stimulation of the INF-γR and STAT1 signaling pathways, indicating phenotypic changes towards the activation of Th1 immunity and M1-type polarization.

### 3.9. Parasite Numbers in the Peritoneal Cavities

**Figure 9:**
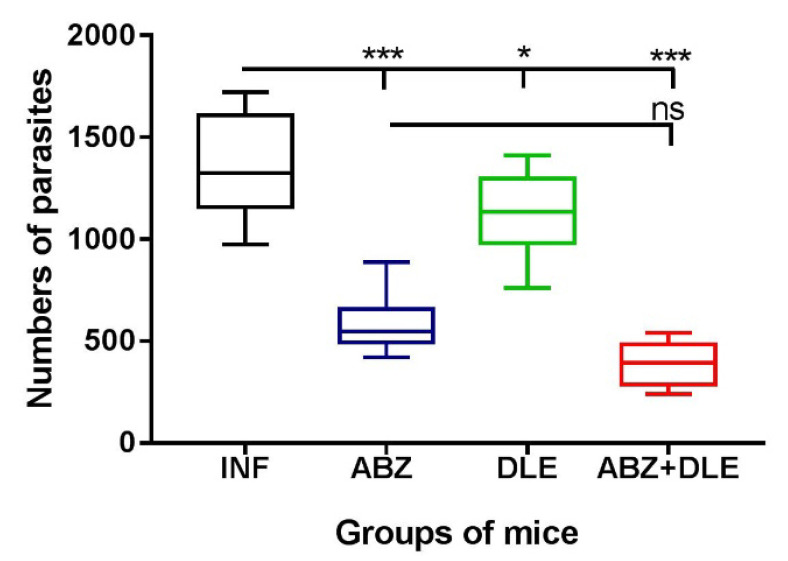
The effect of albendazole (ABZ), DLE, and their combination administration on the numbers of *Mesocestoides vogae* larvae in the peritoneal cavities of infected mice. The numbers represent means ± SD of counts from 14 mice in each group from two experiments. Significantly different counts in comparison with the infected untreated group are indicated as * *p* < 0.05 and *** *p* < 0.001, ns—not significant.

The metacestode (larval) stage of *M. vogae* proliferates asexually, predominantly in the peritoneal cavity of hosts [26,30]. In the present study, we also evaluated the effects of therapy with ABZ and DLE on the reduction in larval numbers on Day 25 p.i./1 post-therapy (Figure 9). In comparison with infected mice (1352.7 ± 225), ABZ significantly reduced larval counts (581.2 ± 65, *p* < 0.001), but the highest reduction was observed after combined treatment with ABZ and DLE (389.2 ± 119, *p* < 0.001), likely due to the specific effect of DLE components. Therapy with DLE resulted in a slight reduction in larval counts (1094.0 ± 201), probably because of the stimulation of the antiparasitic Th1 type of immunity described in the previous paragraphs.

### 3.10. In Vitro Experiments on Adherent Peritoneal Macrophages/Monocytes from Infected Mice

**Figure 10:**
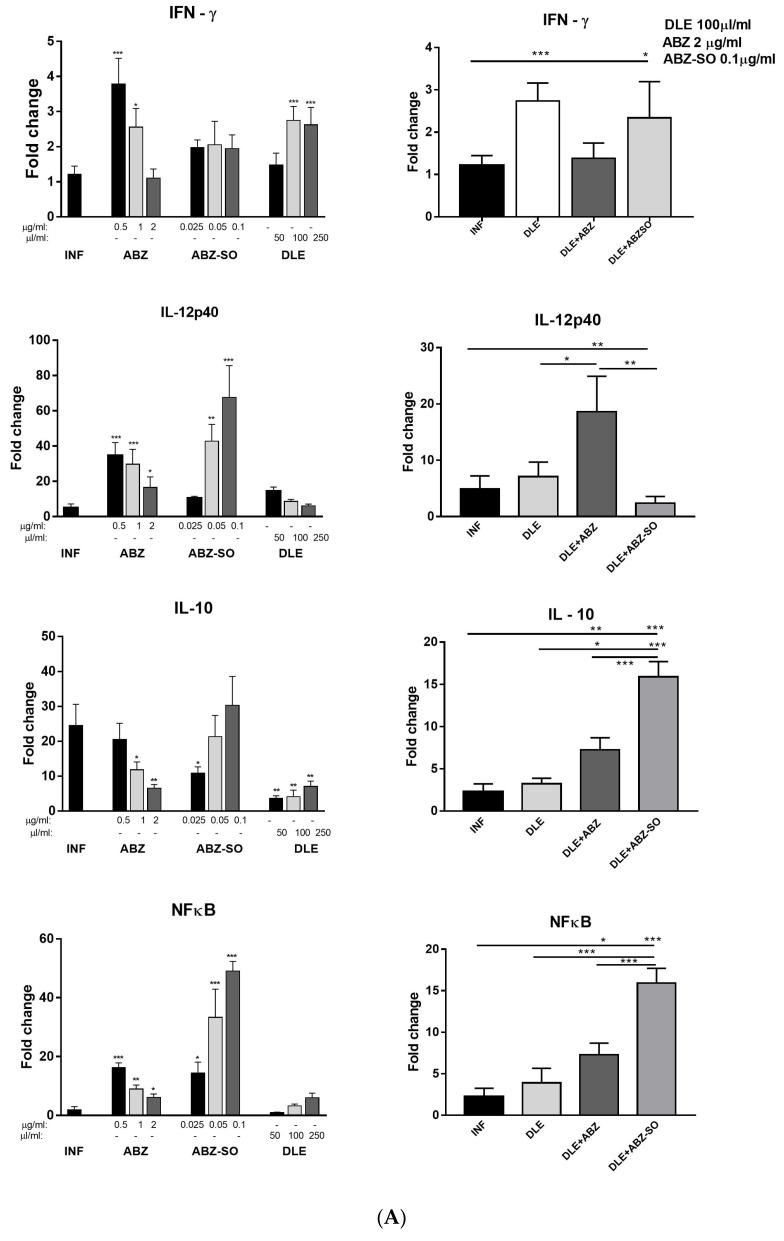

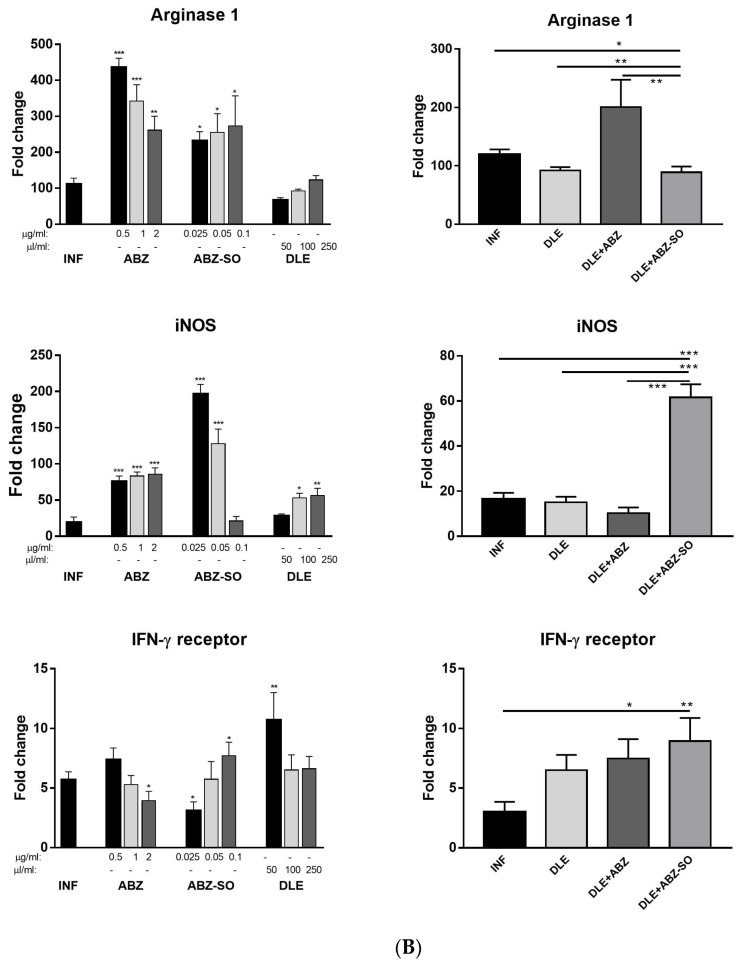
(**A**). Dose-dependent effects of ABZ, ABZ-SO, DLE, and their combinations on relative mRNA expression of genes for cytokines IFN-γ, IL-12p40, IL-10, and NF-κB in adherent peritoneal macrophages/monocytes (1 × 10^6^ cells/well) after 72 h of cultivation ex vivo. Each drug concentration was tested in triplicates and relative gene expression was calculated via the 2^−∆∆Ct^ method, using data for adherent cells from healthy mice as the calibrator. Significantly different values between infected untreated and treated cell samples are indicated as * *p* < 0.05, ** *p* < 0.01, and *** *p* < 0.001. (**B**). Dose-dependent effects of ABZ, ABZ-SO, DLE, and their combinations on relative mRNA expression of genes for selected macrophage markers: arginase 1, iNOS, and IFN-γ receptor in adherent peritoneal macrophages/monocytes (1 × 10^6^ cells/well) after 72 h of cultivation ex vivo. Each drug concentration was tested in triplicates and relative gene expression was calculated via the 2^−∆∆Ct^ method, using data for adherent cells from healthy mice as the calibrator. Significantly different values between infected untreated and treated cell samples are indicated as * *p* < 0.05, ** *p* < 0.01, and *** *p* < 0.001.

We showed in the previous paragraphs that the administration of ABZ and DLE significantly influenced lymphoid and myeloid cells, and their phenotypes and functions, already at the mRNA level. An in vitro experiment on adherent cells aimed to verify the direct effect of ABZ, its active metabolite albendazole sulfoxide (ABZ-SO), and DLE on the transcription machinery in immune cells. After the termination of therapy, adherent cells from infected mice were pooled and the dose-depended effect of ABZ, ABZ-SO, and DLE on mRNA transcription for selected genes was examined. We confirmed that the tested compounds could modulate gene activity at the mRNA level (Figure 10A,B). The effects of higher doses of ABZ (0.5, 1, and 2 µg/mL) were comparable with the effects of lower doses of ABZ-SO (0.025, 0.05, and 0.1 µg/mL) in dose-dependent kinetics, indicating the important role of ABZ-SO in the final effects of ABZ in vivo. In contrast, DLE molecules only moderately altered the gene´s activity, except for the IFN-γ receptor and IFN-γ cytokine. The significant downregulation of mRNA expression for tested genes was found in the cells incubated with DLE in combination with either ABZ or ABZ-SO (at the highest tested concentrations, right panel), suggesting that DLE components act specifically in a dose-dependent way and can modulate the interactions of ABZ and ABZ-SO with ribonucleic acids.

### 3.11. Pharmacokinetic Analysis of ABZ Metabolites in Plasma of Mice

**Figure 11:**
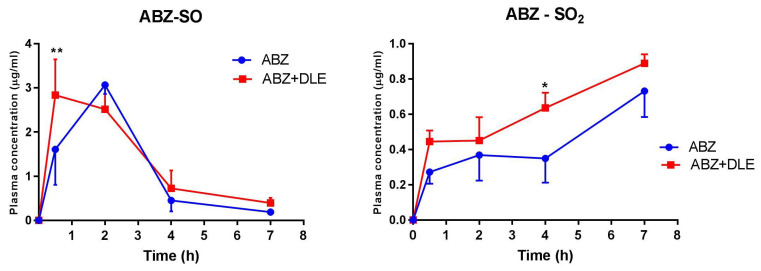
Pharmacokinetic analysis of plasma concentrations (μg/mL) of albendazole metabolite albendazole sulfoxide (ABZ-SO) and albendazole sulfone (ABZ-SO_2_) from the experiment with the same experimental designs was determined via the LC-MS chromatographic method. Significantly different values between infected paired values in each time interval are indicated as * *p* < 0.05 and ** *p* < 0.01.

Finally, in the third experiment we performed the pilot study in which we determined the plasma levels of albendazole sulfoxide (ABZ-SO) and inactive metabolite albendazole sulfone (ABZ-SO_2_) via the LC-MS chromatographic method. The concentrations were calculated from internal standard mebendazole after 30 min, 2, 4, and 7 h after the termination of therapy, and data are shown in Figure 11. The co-administration of DLE moderately elevated the concentration of ABZ-SO in plasma after 10 doses of ABZ administration. In this group, the peak levels (2.83 ± 0.81 μg/mL) were already detected after 30 min (*p* < 0.01) in comparison with the ABZ-treated group, where the concentration of ABZ-SO was the highest after 2 h (3.07 ± 0.20 μg/mL). Within 7 h after the last dose of drugs, concentrations dropped to 0.396 ± 0.10 μg/mL for the combination group and 0.187 ± 0.04 μg/mL for the ABZ-treated group. Concentrations of ABZ-SO_2_ were nearly ten times lower and were slightly elevated in the ABY+DLE-treated group.

## 4. Discussion

Antiparasitic therapy is the primary tool of infection control which relies on a few effective and relatively safe drugs, of which benzimidazole carbamates (albendazole, mebendazole) are recommended by the WHO for the treatment of metacestode (flatworm) infections in humans. They are also used in veterinary medicine. In the current study, we evaluated the impact of ABZ and DLE administration on the inflammatory responses and larval counts in the peritoneal cavity utilizing the *M. vogae* mouse model, where larvae asexually reproduce despite a robust immune response from the mouse hosts [26,28]. In our study, the highest reduction was observed after combined treatment with both drugs, suggesting that DLE, although being non-larvicidal in vitro (unpublished observations), enhanced the drug´s efficacy via the activation of antiparasitic immunity. The highest reduction in infection in Balb/c mice after combined therapy with ABZ and DLE found in the present study was also observed in our previous study on the ICR strain of mice using different batches of DLE (IMMODIN) [34]. Similarly, a significant reduction in *E. multilocularis* cyst growth after co-administration of non-immune DLE prepared from porcine blood leukocytes (Imunor, ImmunomedicA, a.s., Czech Republic) with ABZ and stimulation of Th1 biased immunity was demonstrated in the study of Dvorožňáková et al. [35].

Myeloid-derived cells with a pro-inflammatory signature play a crucial role in effective antiparasitic immunity during helminth infection. Therefore, we further examined whether ABZ and human non-immune DLE modulated the infection-elicited phenotypes and functions of macrophages/monocytes. The numbers of peritoneal exudate cells in *M. vogae*-infected mice elevated with the progressing infection, reaching approximately 30 × 10^6^ cells on Day 25 p.i. (not shown). CD11b is a standard marker expressed on myeloid/monocytic cell lineage, granulocytes [36], and also B1 cells, which represents the main population in the peritoneum of healthy mice [37]. Co-administration of both drugs significantly reduced the population of CD11b+CD19- myeloid-derived cells, which we further analyzed based on the co-expression of the F4/80 membrane protein and the mouse macrophage marker, as well as the major histocompatibility complex (MHCII) receptor. It was shown that the expression of F4/80 glycoprotein is tightly regulated according to the physiological status of the cells and the precursors of tissue macrophages such as monocytes are known to express less F4/80 than their mature counterparts [38]. In all studied groups, the CD11b+F4/80^mid^MHCII^low/mid^ macrophage/monocyte subpopulations were more abundant than the CD11b+F4/80^mid/high^MHCII^high^ cells, and this population was significantly elevated after DLE and more after combination therapy when compared to infected mice. This finding suggests that combined therapy enhanced maturation and/or preferentially stimulated the accumulation of mature macrophages/monocytes with high antigen-presenting capacity via the MHCII receptor. This was also confirmed by the increased expression of both markers, measured as the mean of fluorescence intensity (MFI). The maturation of macrophages requires the participation of co-stimulatory molecules such as CD80/86. In agreement with our hypothesis, Jimenéz-Uribe et al. [39] demonstrated that these molecules were significantly stimulated in LPS-activated THP-1-like macrophages treated with other human DLE (Transferon^®^). However, the effect of ABZ on these markers has not been evaluated yet. After the termination of therapy (Day 25 p.i.), the proportions of CD3+ T cells elevated after the administration of DLE and the proportions of CD19+ B cells increased after ABZ+DLE therapy, whereas ABZ had a moderate suppressive effect on T cells.

It is well known that macrophage differentiation is plastic, allowing them to adapt and acquire many phenotypes depending on the duration of infection and surrounding stimuli. Macrophages activated by inflammatory stimuli such as IFN-γ, LPS, and TNF-α polarize towards a classical M1-like phenotype associated with Th1 immunity, which is an efficient producer of pro-inflammatory cytokines (IL-1, TNF-α, IL-6, and IFN-γ). Macrophages activated by Th2 cytokines, mostly IL-4 and IL-13, are termed as alternatively activated M2-like macrophages and possess a variable capacity to produce inflammatory cytokines and chemokines (for example, IL-4, IL-13, TGF-β, and IL-10). It all depends on the signal utilized for the activation [40,41,42]. Helminths were recognized as inducers and mediators of alternative macrophage activation [43]. Moreover, M2 macrophages express high levels of intracellular proteins FIZZ-1, Ym-1, and arginase 1 which metabolize arginine to collagen precursors, in contrast with inducible nitric oxide synthase (iNOS) converting arginine to NO in M1 macrophages [23,44]. In our study, peritoneal cells from infected mice had significantly upregulated genes for M2-type markers compared to healthy mice. A similar elevation of transcripts for arginase 1, FIZZ-1, and Ym-1 was detected previously in peritoneal exudate cells in a permissive ICR strain of mice with *M. corti* infection [26]. In addition, arginase 1 expression was enhanced in peritoneal myeloid cells in mice with *E. granulosus* infection, which promoted immune evasion by inhibiting the CD4+ T cell receptor functions [45]. We found moderate downregulation of M2 markers and iNOS in peritoneal myeloid cells after ABZ therapy on the transcriptional level. The molecules present in DLE used in our study seemed to suppress the transcription of genes for M2 markers, whereas they upregulated genes for iNOS. It is of note that DLE co-administration significantly modulated ABZ-induced effects on gene activity. Taking these together, combined therapy exerted a positive immunomodulatory effect by reducing the proportions of M2 while elevating M1 macrophage phenotypes. Anti-inflammatory M2 macrophages during metazoan infections serve to dampen immunopathology and sepsis and contribute to wound healing through fibrosis [23]. Therefore, the balanced M1/M2 proportions are considered beneficial to the hosts.

Metacestode growth is controlled by the coordinated cooperation between macrophages and T cells, which is mediated by cytokines, chemokines, and other mediators secreted by various cell types. Our study also examined the expression of selected cytokines in peritoneal cells on mRNA level and their concentrations in peritoneal exudates. Lymphocytes represented more than 30% of PECs and were an important source of all examined cytokines. Infection also elicits a relatively high number of eosinophils (personal observations) which can secrete IL-10 and IL-6 cytokines [46]. ABZ administration moderately downregulated gene transcription for cytokines IFN-γ, TNF-α, IL-12p40, IL-6, IL-4, IL-10, and TGF-β in correlation with their lowered concentrations, except for the elevation of TNF-α. It is known that ABZ induces microtubule destabilization in the parasites and the host´s cells to which it binds with a much lower affinity. This effect could interfere to a certain extent with mRNA transcription and translation processes leading to decreased protein secretion, as was demonstrated, for example, with interferon type I in normal and cancer cells [47].

In the patients with serious metacestode infections caused by *E. granulosus* [48] or *E. multilocularis* [49], successful benzimidazole chemotherapy resulted in the stimulation of gene expression in IL-12p40, IFN-γ, and TNF-α in peripheral blood mononuclear cells. On the contrary, high levels of IL-4 and IL-10 mRNAs were found in the patients in whom therapy failed. In our study, DLE administration significantly stimulated gene activity and the secretion of INF-γ and IL-12, suppressed IL-10 and TGF-β, and reduced IL-4 concentration. This indicates the attenuation of Th2 and the T regulatory type of immunity in correlation with a moderate reduction in parasites. A similar positive effect of DLE on Th1 stimulation was demonstrated in the murine model of tuberculosis treated with antibiotics, in combination with DLE [50]. The important observation of our study was further elevation of IFN-γ secretion and persisting low levels of IL-10 and TGF-β after ABZ+DLE therapy. The IL-10 signaling pathway primarily regulates macrophage and dendritic cells and selectively inhibits the transcription of genes activated during inflammation [51]. The elevated IL-10 cytokine expression was associated with progressing *M. vogae* metacestode proliferation described in our previous study [26]. Nevertheless, ABZ slightly suppressed the DLE-induced effect on IL-12p40. This cytokine is primarily produced by inflammatory macrophages, dendritic cells, and neutrophils, and has a pivotal role in initiating immunity to pathogens [52]. It was shown that the murine resistance to *M. corti* infection in C56/BL mice is dependent on IL-4, as the depletion of this cytokine resulted in increased metacestode proliferation [53]. In their early study, Jenkins et al. [54] suggested that TNF-α plays a role in the impaired macrophage functions during *M. corti* infection, and high proportions of TNF-α-secreting cells in the livers infected with *E. multilocullaris* metacestodes correlated with the active progressing disease [55]. We assume that components present in DLE downregulated the Th2/Treg type of cytokines, stimulated Th1 cytokines on the transcriptional level, and were able to compromise for the negative effects of ABZ on Th1 cytokines. Thus, the observed cytokine profiles after combined therapy seem to contribute the most efficiently to the reduction in parasite numbers and the overall immunopathology. The nuclear factor kappa B (NF-κB) plays a critical role in mediating responses to a remarkable diversity of stimuli and is a powerful orchestrator of the immune response [56]. We showed that it was highly involved in the most profound modulation of immunity after combination therapy.

The expression of iNOS, which generates nitric oxide (NO) by macrophages and, to a lesser extent, by other cells, is triggered by IL-12 and IFN-γ [44]. Unstimulated peritoneal cells from infected and treated mice produced low NO levels after ex vivo cultivation. Although DLE stimulated iNOS gene activity in inflammatory PECs in vivo, the production of NO by unstimulated and LPS stimulated cells was suppressed compared with the infected group and ABZ-treated mice. This might indicate that certain components of intraperitoneally administered DLE could decrease the enzymatic activity of iNOS in the cells. Numerous studies have identified the role of transcription factors in directing macrophage polarization. Interferons potentiate macrophage activation typically via a signal transducer and activation of the transcription (STAT1)-dependent pathway, where binding of IFNs to their receptors triggers a cascade of molecular events, resulting in the upregulation of STAT1 and its phosphorylated form binding to IFN target genes [57,58]. In our study, peritoneal adherent CD11b+CD19- macrophages/monocytes morphologically formed two distinct populations and their proportions were different in individual groups. The co-existence of small and large macrophage subsets was demonstrated in the peritoneal cavity of healthy mice which differed in the expression of F4/80 and MHCII receptors, their morphology, functions, and origin [59], and their proportions can be dramatically changed in response to infections. Immunoreactivity to iNOS, STAT1, and arginase 1 was seen nearly equally in both cell types; therefore, we assume that the smaller round cells could be by their origin monocytes which infiltrated the peritoneal cavity during infection whilst spindle-like cells are phenotypically peritoneal macrophages.

Recently, we showed that STAT1 gene expression in PECs was gradually downregulated while transcript levels for STAT3, STAT6, and IL-10 were markedly elevated within the course of *M. vogae* infection in mice [28]. The binding of IL-10 activates a cascade of molecules including the STAT3 factor required for the anti-inflammatory effects of IL-10 [51]. STAT6 is required to mediate responses to IL-4 and Th2 cell development [60]. In the present study, the gene expression of the IFN-γ receptor and STAT1 were highly upregulated, whereas STAT3, STAT6, and IL-10 mRNA transcripts were significantly decreased after ABZ+DLE therapy. This was in correlation with the elevation of Th1 cytokines and low levels of IL-10, TGF-β, and IL-4 in exudates, as well as the downregulation of M2 macrophage markers. Furthermore, data indicate that DLE components are the potent inducers of the STAT-1 signaling axis on the mRNA level, and their co-administration could partially abolish the suppressive effects of ABZ. To demonstrate that ABZ, ABZ-SO, and DLE can directly interact with mRNAs and modulate the transcription activity of genes for cytokines and macrophage markers, isolated adherent peritoneal macrophages from infected mice were treated for 72 h with different concentrations of drugs. Data on the direct effects of ABZ and ABZ-SO on immune cells are limited. Ramirez et al. [61] showed that ABZ and ABZ-SO arrested cell proliferation in metaphase and increased the frequency of micronuclei in in vitro treated lymphocytes from humans. Here, we showed that ABZ and its metabolite ABZ-SO differentially modulate the transcription of selected genes in a concentration-dependent way. The low water solubility of ABZ and its conversion to active ABZ-SO in the livers results in the relatively low and varying concentrations of individual compounds in plasma and consequently in the peritoneal cavity. The DLE-induced effect on m-RNA levels was most pronounced on IFN-gamma receptor and IL-10, similarly to what was observed in vivo. However, gene transcription profiles were different when DLE was co-cultivated with either ABZ or ABZ-SO, indicating the complexity of interactions with the nucleic acids and other cell compartments, the situation which occurs in vivo during infection and therapy.

A pharmacokinetic analysis of plasma concentrations of ABZ-SO and inactive metabolite ABZ-SO_2_ revealed that peak levels of ABZ-SO appeared much earlier after co-administration of DLE than after drugs given alone, and persisted in slightly higher levels up to 7 h. Similar concentrations of ABZ-SO in the plasma of patients with other flatworm infection neurocysticercosis receiving 30 mg/kg of ABZ for 7 days were detected via the LC-MS/MS method [62]. It seems that DLE probably contributed to the final efficacy of ABZ and modulated gene transcription also by influencing the kinetic of ABZ metabolism in the liver.

## 5. Conclusions

In this study, a comprehensive Th1 promoting DLE activities on various types of immune cells was demonstrated in the peritoneal cavities of *M. vogae*-infected mice, probably as the result of numerous components of non-protein and protein components’ character, including the transfer factor. These molecules seemed to modulate cell differentiation, the expression of selected macrophage receptors, and signaling pathways towars dampening the excessive processes or normalization of suppressed processes already on the molecular level in vivo. Our results also indicate that the modulatory effects of individual DLE components on the imbalanced immune system can enhance the antiparasitic efficacy of the chemotherapy drug, in our case ABZ, and consequently reduce its undesired suppressive effects on the immune system. Thus, the combined therapy with human DLE and ABZ was shown to be more effective than single pharmaceuticals in alleviating parasite-induced immunosuppression by activating the Th1 type of cytokines, the IFN-γ receptor/STAT1 signaling pathway, and M1 macrophage polarization. In parallel, this effect was accompanied with the suppression of Th2/Treg types of cytokines and the downregulation of genes for STAT3, STAT6, IL-10, and TGF-β. The immunomodulatory activities of ABZ, ABZ-SO, and DLE on the peritoneal cells in infected mice seem to result from the interactions of various drug concentrations with ribonucleic acids, or other cell compartments, as was demonstrated by in vitro study. DLE probably contributed to the final efficacy of ABZ and modulated gene transcription also by influencing the kinetic of ABZ metabolism in the liver.

## Data Availability

Data are contained within the article.

## Supplementary Materials

The following supporting information can be downloaded at https://www.mdpi.com/article/10.3390/pharmaceutics15020541/s1, Table S1: List of oligonucleotides and their sequences used in RT-PCR reactions.
