# Supplementary material for: Co-Treatment with Human Leukocyte Extract and Albendazole Stimulates Drug’s Efficacy and Th1 Biased Immune Response in Mesocestoides vogae (Cestoda) Infection via Modulation of Transcription Factors, Macrophage Polarization, and Cytokine Profiles
Source: Pharmaceutics. 2023 Feb 6;15(2):541. doi: 10.3390/pharmaceutics15020541 (PMC9962889; doi:10.3390/pharmaceutics15020541)
Supplement: Supplementary file 1 [file pharmaceutics-15-00541-s001.zip › pharmaceutics-2080042-supplementary.pdf]

pharmaceutics

Supplementary Materials

Co-Treatment with Human Leukocyte Extract and Albendazole Stimulates Drug's Efficacy and Th1 Biased Immune Response in *Mesocestoides vogae* (Cestoda) Infection via Modulation of Transcription Factors, Macrophage Polarization, and Cytokine Profiles

Table S1. List of oligonucleotides and their sequences used in RT-PCR reactions.

Gene	Orientation	Sequence
β-actin	forward	5′-ACCAACTGGGACGACATGGAGAAAATC-3′
	reverse	5′-GTAGCCGCGCTCGGTGAGGATCTTCAT-3′
IL-6	forward	5′-ATGAAGTTCCTCTCTGCAAGAGACT
	reverse	5′-CACTAGGTTTGCCGAGTAGATCTC
IL-12p40	forward	5′-GGA AGC ACG GCA GCA GAT TA-3′
	reverse	5′-AAC TTG AGG GAG AAG TAG GAA TGG-3′
IFN-γ	forward	5′-TCA AGT GGC ATA GAT GTG GAA GAA -3′
	reverse	5′-TGG CTC TGC AGG ATT TTC ATG-3′
TNF-α	forward	5′-GGCAGGTCTACTTTGGAGTCATTGC-3′
	reverse	5′-ACATTCGAGGCTCCAGTGAATTGGG-3′
IL-4	forward	5′-TCAACCCCCAGCTAGTTGTC-3′
	reverse	5′-TTCAAGCATGGAGTTTTCCC-3′
TGF-β	forward	5′-TGACGTCACTGGAGTTGTACGG-3′
	reverse	5′-GGTTCATGTCATGGATGGTGC-3′
IL-10	forward	5- CCA AGC CTT ATC GGA AAT GA-3′
	reverse	5′-TTC TCA CCC AGG GAA TTC A-3′
STAT1	forward	5′ - CTGAATATTTCCCTCCTGGG -3′
	reverse	5′- TCCCGTACAGATGTCCATGAT -3′
IFN-γ receptor	forward	5′-CTC TGC TGT GCG GGT GTA-3′
	reverse	5′-CCA CAC AGC TTC CTC TTG CT-3
Arginase-1	forward	5′- CAGAAGAATGGAAGAGTCAG -3′
	reverse	5′- CAGATATGCAGGGAGTCACC -3′
FIZZ-1	forward	5′- GGT CCC AGT GCA TAT GGA TGA GAC CAT AG-3′
	reverse	5′- CAC CTC TTC ACT CGA GGG ACA GTT GGC AGC-3′
Ym-1	forward	5′- TCA CAG GTC TGG CAA TTC TTC TG-3′
	reverse	5′-TTT GTC CTT AGG AGG GCT TCC TC-3′
iNOS	forward	5′-GCCTCATGCCATTGAATTCATCAACC-3′
	reverse	5′- GAGCTGTGAATTCCAGAGCCTGAA-3′
STAT3	forward	5′-GAAGCCGACCCAGGTGC-3′
	reverse	5′-GTCACGTCTCTGCAGCTTCT-3′
STAT6	forward	5′- GAGTTCCTGGTCGGTTCAGA-3′
	reverse	5′- GCTCTCCAAGGTGCTGATGT-3′
NFκB p65	forward	5′-GACCAACAATAACCCCTTTCAC-3′
	reverse	5′-GTTTGAGATCTGCCCTGATGG-3′
